# Nature's Solution to *Aedes* Vectors: *Toxorhynchites* as a Biocontrol Agent

**DOI:** 10.1155/2024/3529261

**Published:** 2024-06-21

**Authors:** Punya Ram Sukupayo, Ram Chandra Poudel, Tirth Raj Ghimire

**Affiliations:** ^1^ Department of Zoology Bhaktapur Multiple Campus Tribhuvan University, Bhaktapur, Nepal; ^2^ Central Department of Zoology Tribhuvan University, Kathmandu, Nepal; ^3^ Molecular Biotechnology Unit Nepal Academy of Science and Technology (NAST), Lalitpur, Nepal; ^4^ Department of Zoology Tri-Chandra Multiple Campus Tribhuvan University, Kathmandu, Nepal

## Abstract

This review summarizes the predatory potential of *Toxorhynchites* mosquitoes as biological control agents for *Aedes* vectors. A single larva can consume hundreds of mosquito larvae during its development, with preference for larger prey and higher consumption rates at higher prey densities. Studies suggest *Toxorhynchites* can significantly reduce *Aedes* populations. Beyond direct predation, they may indirectly influence prey behavior and adult mosquito lifespan. Despite the demonstrably positive effects of *Toxorhynchites* species as biocontrol agents, there are acknowledged limitations that require further investigation. These limitations include potential variations in effectiveness across diverse habitats and mosquito developmental stages. Additionally, long-term ecological sustainability and potential ramifications warrant further research. Future efforts should prioritize optimizing rearing and release strategies to enhance effectiveness. Exploring the potential for combined control methods with other biocontrol agents or traditional methods is also crucial. Finally, investigating the influence of environmental factors on predation rates can further refine and optimize the application of *Toxorhynchites* in mosquito control programs.

## 1. Introduction

Mosquitoes are notorious vectors for a multitude of diseases worldwide, as the genera *Aedes*, *Anopheles*, and *Culex* are important vectors of mosquito-borne diseases [1]. Among them, *Aedes* spp. are vectors for many arboviruses. The two major species, *Aedes aegypti* (Linnaeus 1762) and *Aedes albopictus* (Skuse 1894), are related to emerging or reemerging infectious diseases resulting serious public health concerns [2]. *Aedes* mosquitoes are diurnal, target both animals and humans for blood meals, and have earned notoriety for their capacity to transmit over 20 viruses and filarial worms, including those with severe implications to human health [3]. Two species *Aedes aegypti* and *Aedes albopictus* were originated from the African continent and Southeast Asian forests, respectively. They are proficient vectors of chikungunya virus, dengue virus, yellow fever virus, and zika virus, inflicting significant health repercussions and economic losses worldwide.

Dengue fever, the most prevalent mosquito-borne disease, has seen a tenfold increase in reported cases globally between 2000 and 2019 [4]. This translates to more than 3.9 billion people in over 129 countries being affected, with over 40,000 deaths every year [5]. While Asia carries about 70% of world's disease burden, the Americas, Southeast Asia, and the Western Pacific are hardest hit. The situation worsens as dengue fever creeps into new areas like Europe, while existing regions experience intense regular outbreaks [6]. According to the WHO, the Americas experienced the most cases in 2023 with over 4 million reported, highlighting this growing threat. Southeast Asia remains a major hotspot with countries like Thailand and Vietnam experiencing high numbers of casualties [4]. Africa also faces a rising burden with outbreaks in 15 countries. Similarly, the Eastern Mediterranean region is experiencing an increase, with nine countries regularly facing outbreaks. While Europe is not considered endemic, a few countries like Italy, France, and Spain did report outbreaks in 2023 [4]. Thus, this rise in mosquito-borne diseases is of global concern. Chikungunya, another *Aedes*-borne disease, saw major outbreaks in East Africa, the Indian Ocean (2005-2006), and the Americas and Oceania (2013-2014) [7–9]. Zika also caused major outbreaks in the Americas (2015-2016) [9]. Moreover, yellow fever has reemerged with recent epidemics in sub-Saharan Africa and Brazil [10].

The primary strategy to minimize the risk of mosquito-borne infections involves reducing mosquito populations [11]. The emergence of insecticide resistance and the detrimental effects of conventional insecticides on the environment have fueled a growing interest in biological control methods for mosquito vector management [12]. Various biocontrol agents from the insect orders Diptera, Odonata, Coleoptera, and Hemiptera have been explored globally to control mosquito populations [13]. From the outset, scientists have recognized the value of utilizing the natural predator-prey relationships within the environment for effective mosquito control. These natural predators including juvenile fish, Odonata larvae (dragonfly and damselfly nymphs), and mosquito larvae offer a sustainable and environmentally friendly approach to control mosquito [14–16]. A fascinating paradox exists among these predators: the *Toxorhynchites* mosquito. These giants of the mosquito world, nicknamed “elephant mosquitoes” or “mosquito eaters,” possess a proboscis specifically adapted for nectar, not blood. *Toxorhynchites* mosquitoes, primarily tropical dwellers found in the lush forests of equatorial and tropical regions, have a distribution that generally falls within a band between 35° north and 35° south latitude [17]. However, there are a few exceptions. Some species, like *Toxorhynchites rutilus*, has adapted to the temperate zones of the Northern Hemisphere [18] with a wider range within North America, stretching from Mexico all the way to the Atlantic coast [19, 20]. While a few species have ventured beyond the tropics, Southeast Asia remains a hotspot for *Toxorhynchites* diversity, boasting as many as 24 documented species [21].

The larvae of *Toxorhynchites* mosquitoes hold immense potential for mosquito control [22, 23]. These larvae are exceptional predators throughout their development, particularly adept at preying on mosquito larvae of public health significance, such as *Aedes aegypti* (L.), *Aedes albopictus* (Skuse), and *Culex quinquefasciatus* [24, 25]. Interestingly, *Toxorhynchites* often lay eggs in the same containers favored by *Aedes aegypti* and *Aedes albopictus* [26, 27]. A single *Toxorhynchites* larva can devour a staggering number of prey larvae—up to several thousand during their whole larva stage [22]. Their predatory prowess extends beyond direct consumption. *Toxorhynchites* larvae exhibit a fascinating behavior known as compulsive killing, where they kill mosquito larvae but leave them uneaten, especially before pupation [28]. Additionally, they can indirectly influence prey development, further hindering their population growth [29]. Consequently, *Toxorhynchites* larvae represent a promising model for *Aedes* mosquito control. Therefore, the aim of the current review is to analyze the role of *Toxorhynchites* larvae in the control of *Aedes* mosquito around the world.

## 2. Methods

**Figure 1:**
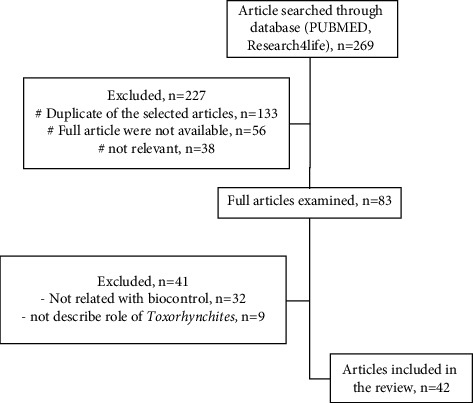
Research design.

We conducted a comprehensive search for articles in Science Citation Index (SCI)-indexed journals, focusing on *Aedes* mosquito control and the potential of *Toxorhynchites* as a biocontrol agent. Utilizing PubMed and Research4Life, we searched using the keywords “*Aedes* control” and “*Toxorhynchites*” to capture relevant strategies and the specific role of *Toxorhynchites*. The initial search yielded 269 articles. To maintain high scientific rigor and align with the review's objectives, we applied specific inclusion/exclusion criteria related to *Aedes* control and *Toxorhynchites*' biocontrol role. This resulted in a final selection of 42 articles for detailed analysis (Figure 1). To deeply understand existing research, a standardized data extraction method was employed, collecting information on study design, methodology, key findings, and limitations from each article (Supplementary Table 1).

### 2.1. Inclusion Criteria

- Studies investigated the use of *Toxorhynchites* larvae or adults for controlling *Aedes* mosquito populations.
- Studies evaluated the effectiveness of *Toxorhynchites* in reducing *Aedes* mosquito breeding or biting rates.
- Studies conducted in laboratory or field settings that explored the predatory behavior of *Toxorhynchites* toward *Aedes* mosquito larvae.
- Studies published in English in the last 51 years (1972 to 2023).

### 2.2. Exclusion Criteria

- Studies focused on *Toxorhynchites* for purposes other than *Aedes* mosquito control (e.g., their biology, taxonomy, and distribution).
- Studies only mentioned *Toxorhynchites* in passing without any data on their use in biocontrol.
- Studies published in journals that are not SCI-indexed.
- Studies published in languages other than English (unless translations were available).

## 3. Results

### 3.1. Global Distribution

*Toxorhynchites* mosquitoes boast an impressive global presence, extending across continents from Asia (Indonesia, India, and Thailand) to Africa (Tanzania) and North America (Florida, USA, and Mexico). *Toxorhynchites splendens* notably demonstrated a wide distribution, encompassing Bangladesh, Nepal, Myanmar, and Sri Lanka (Table 1). Building on this diversity, Table 2 highlights 13 potential *Toxorhynchites* species that could be recruited to the fight against mosquitoes, demonstrating the genus's promising role in biocontrol solutions.

### 3.2. *Toxorhynchites* Record from Nepal

Only one species of *Toxorhynchites* (*Tx. splendens*) has been documented in Nepal. *Toxorhynchites splendens* was first recorded in 1956 by Peter and Dewar [64]. It has been documented in Sunsari, Rupandehi, Jogikuti, Sindhuli, and Khuntpani in Nepal, as reported by Darsie and Pradhan in 1990 [65]. It has also been reported from the Banke district by Darsie et al. in 1996 [43]. However, there have been no published records of *Toxorhynchites* in Nepal after 1996.

### 3.3. Promising Species for Biocontrol

Several *Toxorhynchites* species show high potential as biological control agents for mosquito populations, particularly *Aedes aegypti*, and the vector of dengue hemorrhagic fever. Their key strengths lie in their predatory efficiency and eco-friendly nature. Many *Toxorhynchites* larvae are voracious predators, consuming hundreds of mosquito larvae per individual throughout their lifespan [44, 45]. Field trials documented reductions in *Aedes* populations by up to 83% after *Toxorhynchites* introductions [46]. Unlike chemical insecticides, *Toxorhynchites* pose minimal risk to nontarget organisms and the environment [36, 47]. This makes them particularly attractive for areas with environmental, biodiversity, and other concerns.

#### 3.3.1. *Toxorhynchites splendens*

*Toxorhynchites splendens* larvae are known to be efficient predators, consuming a significant number of prey larvae daily. Studies show impressive predation rates in the laboratory. For example, single third-instar larvae in West Bengal, India, have been observed to consume over 50 *Ae. albopictus* larvae daily [44]. Similar results were observed in Okinawa, Japan, where research recorded that fourth-instar *Tx. splendens* larvae could consume as many as 55 *Ae. albopictus* larvae daily, while third-instar larvae exhibited a lower predation rate, consuming around 20 larvae per day [45]. Another study in Sri Lanka demonstrated that third- and fourth-stage larvae of *Tx. splendens* could devour one *Ae. albopictus* larvae in about 30 minutes [46]. Even with some studies showing lower predation rates [48], the effectiveness of *Tx. splendens* translates encouragingly from laboratory to real-world settings. Semifield experiments showed significant reductions in *Ae. albopictus* larvae [44]. Field trials documented up to 83% decline in *Aedes* populations following *Tx. splendens* introductions [46]. Additionally, a negative correlation between *Tx. splendens* and *Ae. albopictus* larvae in ovitraps was observed [26]. Even monthly adult releases effectively reduced target mosquito broods in containers [49]. However, factors such as container type and prey density (offering 10 to 50 prey individuals per predator) influenced *Tx. splendens* predation rates, with horizontal containers with wide openings being more suitable [48].

Additionally, experiments explored *Tx. splendens*' feeding activity in the presence of alternative food, reaffirming its role as a mosquito larvae predator. They exhibit a clear preference for consuming mosquito larvae, and their consumption is inversely proportional to the search area and directly proportional to prey density (the number of prey given) [44, 50]. Interestingly, fourth-instar *Tx. splendens* larvae might also kill prey without consuming them [45]. Researchers also investigated *Tx. splendens*' behavior to optimize control programs, revealing hunting preferences for *Aedes aegypti* over *Aedes albopictus*, with predation increasing with prey density [51]. *Tx. splendens* larvae prefer *Aedes* larvae of the respective stages [45, 51], with a preference for *Ae. aegypti* larvae over *Ae. albopictus* and *Anopheles sinensis* due to more active movements [47]. However, *Tx. splendens* larvae exhibited a slightly lower predation ability for *Ae. aegypti* compared to *Culex quinquefasciatus* (10.6 vs. 12 larvae/day) [50].

Research suggests *Tx. splendens* larvae may even kill prey without consuming them. While this highlights their effectiveness, the long-term ecological implications need thorough investigation. Furthermore, complete eradication of target mosquito populations might not be achievable solely with *Tx. splendens* releases [46]. Integrated control programs combining *Tx. splendens* with other methods may be necessary. However, potential ecological impacts and limitations on complete eradication necessitate a cautious approach with *Tx. splendens* as a biocontrol agent.

#### 3.3.2. *Toxorhynchites amboinensis*

Laboratory studies reported that a single predator larva can consume more than 230 prey larvae on average throughout its larval stage at lower prey density (20 *Ae. aegypti* larvae/200 ml water/*Toxorhynchites* larva). Interestingly, it moves more than 350 times at higher prey density (*Ae. aegypti* larvae/200 ml water/*Toxorhynchites* larva) [50] regardless of the prey species offered (*Ae*. *aegypti*, *Ae. albopictus*, and *Cx. quinquefasciatus*). These findings highlight *Tx. amboinensis* as a potential predator for a variety of mosquito larvae, such as *Aedes aegypti*, *Aedes albopictus*, and *Culex quinquefasciatus*. The fourth-instar larvae were found to be the most predacious, with compulsive predation observed during the late stage of this instar [30], confirming its predatory efficacy and pinpointing the fourth-instar larvae as the most effective stage. Interestingly, these studies also revealed a preference for mosquito larvae by *Tx. amboinensis*, with higher predation rates at higher prey densities. It suggests *Tx. amboinensis* can be a valuable component of an integrated mosquito control strategy, potentially offering both environmental and health benefits by reducing reliance on chemical insecticides due to its preference for mosquito larvae and increased predation at higher prey densities.

Field trials investigated *Tx. amboinensis* as a promising biological agent for mosquito control. Studies in New Orleans [31] demonstrated significant reductions (up to 45%) in *Ae. aegypti* densities following weekly releases of *Tx. amboinensis* larvae. This predator's effectiveness was further amplified when combined with reduced insecticide use. When integrated with Malathion, mosquito control reached up to 96% compared to 29% with Malathion alone [32]. Interestingly, increasing the number of *Tx. amboinensis* released did not yield additional control, suggesting researchers could develop optimized release strategies [31]. However, the success rate of *Aedes* spp. control by *Tx. amboinensis* depends greatly on the type of habitat, as the mean overall reduction of *Aedes* by the introduction of *Tx. amboinensis* was recorded as 22% in tins and 63% in tires over a 10-month period in Wailoku Village and Yanuca Island [33]. Habitat suitability assessments were therefore essential for determining the feasibility of *Tx. amboinensis* as a control agent in specific locations. Overall, *Tx. amboinensis* demonstrated promise as a biological control agent for vector mosquito species. Its effectiveness as a predator, potential for integration with reduced insecticide use, and environmentally friendly approach make it a valuable candidate for further development. However, successful implementation requires careful consideration of release strategies, habitat suitability, and ecosystem dynamics. Future research should focus on optimizing these aspects to maximize the potential of *Tx. amboinensis* for sustainable mosquito control.

#### 3.3.3. *Toxorhynchites moctezuma*

A dose-dependent effect, with more *Tx. moctezuma* larvae leading to a sharper decline in *Ae. aegypti* emergence, was documented in the experiment conducted at the Caribbean Epidemiology Centre in Spain. One or two *Tx. moctezuma* larvae could halt adult *Ae. aegypti* emergence for a week, and five or ten larvae could prevent emergence for up to 16 weeks [37]. Additionally, sustained releases of *Tx. moctezuma* larvae for five months resulted in lower mosquito indices in released villages relative to unreleased ones [38], suggesting long-term effectiveness. *Tx. moctezuma* larvae exhibit minimal cannibalism, but their fourth-instar stage displays compulsive killing behavior [37]. These contrasting traits present both challenges and opportunities for their use as biocontrol agents. The successful suppression of *Ae. aegypti* populations through systematic releases of *Tx. moctezuma* larvae on Union Island [39] further bolstered the case for this biological control method. However, a decline in *Tx. moctezuma*'s effectiveness over time was observed [36], highlighting the need for research on maintaining long-term control. Most studies focused on single-release events or short-term trials [37, 39]. Therefore, large-scale field studies were needed to assess long-term efficacy and ecological impact. Additionally, cost-effective rearing and release strategies were not addressed in the presented studies. Overall, the evidence strongly supports the potential of *Tx. moctezuma* larvae as a powerful biological control agent for *Ae. aegypti*. However, further research is necessary to address limitations like diminishing control over time, develop practical rearing and release methods, and conduct a thorough assessment of potential ecological impacts before widespread implementation.

#### 3.3.4. *Toxorhynchites rutilus*

*Tx. rutilus* larvae emerged as efficient predators in past studies [40, 66]. A single larva could consume or kill nearly 50 mosquito immatures daily, exceeding 300 during development [40]. Another research documented as many as 400 prey larvae during their larval development and also demonstrated killing prey without consuming it [40, 66], which could significantly reduce potential adult *Ae. aegypti* emergence. The fourth-instar *Tx. rutilus* were more likely to kill pupae than the larvae of the same age. Interestingly, they completely ignored the first instar altogether [40]. This preference for larger prey size suggests a potential limitation for *Tx. rutilus* as a biocontrol agent—while powerful; it might not be equally effective against all mosquito life stages. Additionally, studies documented cannibalism occurring in confined spaces with limited resources [66]. Understanding and mitigating these behaviors are crucial for optimizing the effectiveness and sustainability of *Tx. rutilus*-based interventions. However, natural populations of *Tx. rutilus* were often insufficient for mosquito control, necessitating the rearing and release of additional adults. This approach increases complexity and cost. Additionally, the effectiveness of *Tx. rutilus* releases relied on precise timing, requiring releases before mosquito populations surged [41]. Adult dependence on nectar sources added another layer of complexity [41]. Availability of suitable nectar sources can limit their effectiveness in certain areas. While such programs offer potential advantages like dispersal and positive public perception, successful implementation requires careful planning, monitoring, and adaptation to local conditions. A study investigating the combined effects of pyriproxyfen (an insecticide) and *Tx. rutilus* on *Ae. aegypti* found that the combined approach significantly inhibited adult emergence compared to using either method alone [42]. However, further research with larger sample sizes and diverse field conditions is necessary to confirm these findings and assess the long-term efficacy and ecological implications of this combined approach. Overall, *Tx. rutilus* showed promise as a biological control agent, but its limitations necessitate a multifaceted approach. Future research can refine rearing and release strategies, alongside exploring complementary control methods, to maximize effectiveness of *Tx. rutilus* in managing *Ae. aegypti* populations.

#### 3.3.5. *Toxorhynchites brevipalpis*

A detailed analysis conducted at the Department of Biology, University of Notre Dame, Indiana, revealed that predation rates of *Tx. brevipalpis* on *Ae. aegypti* larvae varied with temperature, with the highest predation (*n* = 358) observed at 30–32°C during larval development [27]. Interestingly, *Tx. brevipalpis* also killed prey larvae nearing pupation without consumption [27]. Similarly, combining *Metarhizium brunneum* fungus with *Tx. brevipalpis* larvae resulted in significantly lower *Ae. aegypti* larval survival rates than using either approach alone [35]. However, generalizing these laboratory findings to field applications requires careful consideration of ecological complexities and practicalities. Future research should prioritize field trials to validate the efficacy and sustainability of combined control strategies.

#### 3.3.6. *Toxorhynchites violaceus*

Experiments performed in the laboratory to find the survival rate of fourth-instar larvae of *Ae. aegypti* in the presence of fourth-instar larvae of *Tx. violaceus* showed that although the initial survival rate was 98% in 24 hours, it decreased subsequently, reaching 0% by 192 hours [53]. This suggests a significant increase in the predatory potential of *Tx. violaceus* fourth-instar larvae for consuming *Ae. aegypti* larvae, ultimately leading to the elimination of all *Ae. aegypti* larvae. While this laboratory experiment was promising, the real-world effectiveness of *Tx. violaceus* remained uncertain. Furthermore, field testing is imperative as natural environments introduce complexities that laboratory settings cannot fully replicate.

#### 3.3.7. *Toxorhynchites towadensis*

*Tx. towadensis* exhibited a curious behavior of killing prey without consuming them at high prey densities. While the study offers valuable insights, its generalizability is limited by the artificial setting. Real-world environments present greater complexities in prey availability, habitat structure, and potential interactions with other species. Future research should explore how these factors interact with prey density to provide a more holistic understanding of *Tx. towadensis*' predatory behavior in natural settings. Moreover, the study solely focused on prey density. Investigating the influence of prey type and environmental conditions on *Tx. towadensis*' behavior and development would provide a more comprehensive picture of its potential role in mosquito population control strategies [52].

#### 3.3.8. *Toxorhynchites theobaldi*

*Aedes aegypti* mosquitoes, instead of avoiding predators, preferred oviposition sites with evidence of *Tx. theobaldi* predation (including dead conspecific larvae) due to the increased bacterial abundance by *Tx. theobaldi* feeding activity [54]. The study sheds light on the intricate web of indirect effects within predator-prey interactions, where predation can influence prey behavior through alterations in the microbial community. This suggests a potential double benefit for employing *Tx. theobaldi* as a biocontrol agent: direct reduction of prey populations and the inadvertent attraction of egg-laying mosquitoes to areas where their offspring are more susceptible to predation [54]. However, limitations exist. The laboratory setting might not fully capture the complexities influencing oviposition choices in natural environments. Additionally, the study focused solely on bacterial cues. Future research should explore the specific bacterial strains involved and how they interact with other environmental factors influencing mosquito behavior.

### 3.4. Combined Study of Different Species

An investigation of feeding strategies in five *Toxorhynchites* mosquito species (*Tx. amboinensis*, *Tx. rutilus*, *Tx. theobaldi*, *Tx. brevipalpis*, and *Tx. splendens*) found no significant difference in the average number of strikes needed for a successful capture across all species. However, *Tx. amboinensis* (37 prey/day) and *Tx. brevipalpis* (35 prey/day) captured prey at a significantly higher rate than *Tx. splendens* (27 prey/day), *Tx. theobaldi* (25 prey/day), and *Tx. rutilus* (19 prey/day) [34]. Interestingly, *Tx. theobaldi* exhibited a higher daily consumption rate (18.9 ± 0.97 prey/day) than other predators, even killing prey beyond their immediate needs before pupation [34]. These findings suggest potential variations in feeding strategies among *Toxorhynchites* species. Future research should delve deeper, investigating factors like predatory activity, handling time, and prey type to provide a more comprehensive understanding of these species' feeding strategies. Furthermore, examining if these patterns hold true across all developmental stages, from larvae to adult, is crucial for a complete picture.

### 3.5. Usefulness of Other Insects Other than *Toxorhynchites*

Some other invertebrates such as copepods, dragonflies, and damselflies are also predators of mosquito larvae. Copepods are highly effective predators, capable of significantly reducing larval populations, even achieving complete elimination in some cases [67]. While copepods primarily target mosquito larvae, they also feed on other aquatic organisms, which help to maintain ecological balance in water bodies [68, 69]. However, their ability to control mosquito populations might be reduced because they are not exclusively mosquito predators and may consume other organisms [67, 70]. Additionally, only large copepod species (over 1 mm) can effectively prey on mosquito larvae, limiting the range of species suitable for biocontrol [67]. The impact of copepod introduction on nontarget species and ecosystem dynamics requires careful assessment to avoid unintended consequences [71]. Odonata larvae, encompassing dragonflies and damselflies, are voracious mosquito predators, effectively reducing mosquito populations [13]. Their extended larval development makes them ideal biocontrol agents, allowing for continuous mosquito predation [72]. However, this long development period can be a drawback. Dragonfly larvae in temporary ponds may not survive extended droughts, hindering control effectiveness [73]. Additionally, some Odonata larvae exhibit prey preferences, favoring other aquatic insects over mosquito larvae [74]. The research on copepods, dragonflies, and damselflies suggests they could be useful for mosquito control, but there are also some drawbacks like impact on other organisms and long development times.

*Toxorhynchites* mosquitoes are a promising choice for biocontrol against mosquitoes compared to Copepoda and Odonata (dragonflies and damselflies). They target mosquito larvae specifically, minimizing disruption to the ecosystem. Additionally, their faster life cycle allows for quicker population control compared to Odonata whose development can be slow. These factors make *Toxorhynchites* a compelling option for eco-friendly mosquito management. These studies highlight *Toxorhynchites* species as mosquito predators [27, 33, 41, 44, 48, 50]. Among *Toxorhynchites* species, *Tx. splendens* stands out for its well-documented success in controlling *Aedes* larvae, though effectiveness might vary against *Ae. aegypti*. *Tx. amboinensis* offers a broader range of mosquito prey but requires study on its ecological impact. *Tx. moctezuma* shows promise for long-term suppression but may necessitate frequent releases. *Tx. rutilus* boasts high predation rates but has limitations in target stages and requires careful management to avoid cannibalism. *Tx. brevipalpis* excels in warm climates and might work well with other control methods, but field data are limited. *Tx. violaceus* and *Tx. towadensis* show promise in the laboratory but need real-world testing. While *Tx. theobaldi* offers an intriguing indirect control mechanism, its predatory impact is less direct. However, the potential of many *Toxorhynchites* species remains unstudied, offering a wealth of potential for future mosquito control research.

## 4. Conclusion

Various *Toxorhynchites* mosquito species exhibit promising potential as biological control agents for mosquito populations, particularly *Aedes aegypti*, the vector of dengue hemorrhagic fever. Their key strengths lie in their voracious predatory nature and minimal ecological impact. Species like *Tx. splendens*, *Tx. amboinensis*, and *Tx. moctezuma* have demonstrated significant reductions in *Aedes* populations in both laboratory and field trials. Based on the available data, among *Toxorhynchites* species, *Tx. splendens* stands out for its well-documented success in controlling *Aedes* larvae and *Tx. amboinensis* appears to be the most suitable species for controlling *Aedes* mosquito populations due to its high predation rates, effectiveness against various mosquito larvae, and successful field trials. However, limitations exist, including potential variations in effectiveness across habitats and developmental stages, as well as the need for further research on long-term sustainability and potential ecological ramifications. Future research should focus on optimizing rearing and release strategies, exploring combined control methods, and investigating the influence of environmental factors on predation rates. By addressing these limitations, *Toxorhynchites* species can become a valuable component of integrated mosquito management programs, offering a safe and eco-friendly approach to curbing mosquito-borne diseases.

## Figures and Tables

**Table 1:** Geographical distribution of *Toxorhynchites*.

S.N.	Species	Country	Source
1	*Tx. acaudatus*	Indonesia	[21]
2	*Tx. albipes*	India and Thailand	

3	*Tx. amboinensis*	Indonesia	[21, 30–34]

4	*Tx. auranticauda*	Indonesia	[21]
5	*Tx. aurifluus*	Taiwan	
6	*Tx. bengalensis*	Bangladesh	
7	*Tx. bickleyi*	Thailand	

8	*Tx. brevipalpis*	Tanzania (Africa), United Kingdom, USA	[21, 27, 34, 35]

9	*Tx. coeruleus*	Indonesia	[21]
10	*Tx. christophi*	DPR Korea	
11	*Tx. edwardsi*	India	
12	*Tx. gravelyi*	India and Thailand	
13	*Tx. guadeloupensis*	Brazil	
14	*Tx. inornatus*	Indonesia	
15	*Tx. kempi*	India, Indonesia	
16	*Tx. klossi*	India	
17	*Tx. leicesteri*	Thailand	
18	*Tx. magnificus*	Thailand	
19	*Tx. manicatus*	Taiwan	
20	*Tx. manopi*	Thailand	
21	*Tx. metallicus*	India and Indonesia	
22	*Tx. minimus*	India, Indonesia, and Sri Lanka	

23	*Tx. moctezuma*	Mexico, USA	[21, 36–39]

24	*Tx. quasiferox*	Indonesia	[21]

25	*Tx. rutilus*	Florida (USA)	[21, 34, 40–42]

26	*Tx. speciosus*	Indonesia	[21]

27	*Tx. splendens*	Bangladesh, India, Indonesia, Nepal, Myanmar, Sri Lanka, Malaysia, Philippines, Japan, USA, and Thailand	[21, 26, 34, 43–51]

28	*Tx. sumatranus*	Indonesia	[21]
29	*Tx. sunthorni*	Thailand	

30	*Tx. towadensis*	Florida, Japan	[21, 52]

31	*Tx. tyagii*	India	[21]

32	*Toxorhynchites violaceus*	Brazil	[53]

33	*Toxorhynchites theobaldi*	Brazil, USA	[34, 54]

**Table 2:** Global utilization of *Toxorhynchites* species for biological control.

S. N	Species	Country	Citation
1	*Toxorhynchites rutilus*	Florida (USA)	[20, 55]
2	*Toxorhynchites splendens*	Malaysia, Indonesia, Russia, India, and USA	[26, 29, 44, 47, 48, 50, 56, 57]
3	*Toxorhynchites amboinensis*	Florida, Texas, Louisiana (America), Mexico, Philippines, and Malaysia	[24, 30–32, 42, 48, 58, 59]
4	*Toxorhynchites towadensis*	Florida	[22, 28]
5	*Toxorhynchites moctezuma*	Mexico, USA	[36, 39]
6	*Toxorhynchites brevipalpis*	Tanzania (Africa)	[27, 60]
7	*Toxorhynchites*	Brazil	[61]
8	*Toxorhynchites christophi*	Korea	[62]
9	*Toxorhynchites aurifluus*	Taiwan	[63]
10	*Toxorhynchites manicatus*	Taiwan	[63]
11	*Toxorhynchites theobaldi*	Brazil	[54]
12	*Toxorhynchites violaceus*	Brazil	[53]
13	*Toxorhynchites minimus*	Sri Lanka	[46]

## Data Availability

No primary data were used to support this study. Information presented in this review is based on published papers, which have been duly cited.

## Supplementary Materials

Supplementary Materials Supplementary Table 1. Spreadsheet of extracted data.
