# Supplementary material for: Nature's Solution to Aedes Vectors: Toxorhynchites as a Biocontrol Agent
Source: J Trop Med. 2024 Jun 21;2024:3529261. doi: 10.1155/2024/3529261 (PMC11213640; doi:10.1155/2024/3529261)
Supplement: Supplementary Materials — Supplementary Table 1. Spreadsheet of extracted data. [file 3529261.f1.docx]

**Supplementary files**

Supplementary table 1: Spreadsheet of extracted data

| Citation | Species used | Study design | Methodology | Key finding | Limitation |
| --- | --- | --- | --- | --- | --- |
| Mohamad, and Zuharah, 2014 | *Tx. splendens* | Factorial experiment with container type (4 levels) and water volume (4 levels) | Evaluated the predation rate of *Tx.* *splendens* third instar larvae on *Ae.* *albopictus* larvae in 24 h. | Higher prey density led to more consumption, horizontal containers (tires) saw higher predation | Lab setting limits real-world applicability |
| Zuharah et al., 2015 | *Tx. splendens* | Observational | Examined prey choices by *Tx.* *splendens* by monitoring the behavioral responses of *Aedes aegypti, Aedes* *albopictus*, and *Anopheles sinensis* larvae when exposed to the predator. | *Ae. aegypti* might have a weaker ability to detect predator cues, leading to increased risk of predation | Didn't directly measure predation rate, only suggests vulnerability based on behavior |
| Muhamat et al., 2022 | *Tx. splendens* | randomized | Predation rate and development time were measured | Predation for *Ae. aegypti* and *Cx. Quiquefaciatus* larvae is almost similar | Didn't mention a control group |
| Malla et al.,2023 | *Tx. splendens* | Factorial experiment | Tested feeding activity under varying conditions like search area (water volume), prey density, prey instar stage, and presence of alternative food (*Tubifex*). | Predation rate increased with higher prey density and decreased with larger search area (more water) | Didn't mention details about replication |
| Millado and Sumalde, 2018 | *Tx. splendens* | Choice and No-choice experiment | Offered *Tx.* *splendens* larvae (different instars) prey choices (*Ae. aegypti, Ae. albopictus, Cx. quinquefasciatus*) at varying densities (with/without choice) | *Tx.* *splendens* consumed more *Ae. aegypti* than *Ae. albopictus* across all densities. | Controlled laboratory setting might not reflect real-world feeding behavior. |
| Nyamah et al., 2011 | *Tx. splendens* | Observational | Installation and study using ovitraps | Negative correlation exists between *Tx.* *splendens* and *Ae.* *albopictus* larval populations | Suggested a correlation, not predator-prey interaction |
| Toma and Miyagi, 1992 | *Tx. splendens* | Observational | Individual *Tx.* *splendens* larvae were likely provided with a constant supply of prey throughout their development stages (instars) and the number of consumed prey was recorded daily. | Predation peaked during the fourth instar | Didn't explore reasons behind the observed feeding patterns |
| Wijesinghe et al., 2009 | *Tx.* (*splendens* and *minimus*) | Field experiment | Introduced varying numbers of each predator (larvae/fish) into tanks, monitored *Aedes* larvae reduction after one week | Fish were significantly faster at consuming *Aedes* larvae compared to *Toxorhynchites* | Limited observation period (one week) |
| Miyagi et al., 1992 | *Tx. splendens* | field study | Monthly releases of *Tx.* *splendens* and monitored the impact on target mosquito populations | Effectively controlled target mosquito breeding in artificial containers. | Pilot study with limited scope and duration |
| Focks et al., 1985 | *Tx. amboinensis* | Field experiment | Released *Tx. amboinensis* larvae weekly and compared *Ae. aegypti* densities between treated and untreated areas | Significantly reduced *Ae. aegypti* mosquito | Limited information on baseline mosquito densities |
| Focks et al., 1986 | *Tx. amboinensis* | Field experiment comparing three treatments: * Control * Malathion application alone * Integrated treatment | Monitored *Ae. aegypti* densities | Integrated treatment achieved a significantly higher reduction | Lacked details on the predator species, release strategy, and Malathion application schedule were missing |
| Digma et al., 2019 | *Tx. amboinensis* | Laboratory experiment | *Tx.* amboinensis were reared, their larvae exposed to different prey densities | Predation rate increased significantly with higher prey density | Laboratory setting limits real-world applicability |
| Toohey et al., 1985 | *Tx. amboinensis* | Field experiment | Released *Tx.* amboinensis females and monitored mosquito larvae (*Aedes* & *Toxorhynchites*) in containers placed in release areas | Containers with *Tx.* *amboinensis* had significantly fewer *Aedes* larvae compared to those without | Didn't track adult mosquito populations |
| Uejio et al, 2014 | *Tx. moctezuma* | Observational on natural habitat | Compared mosquito pupae in areas with and without *Tx.* *moctezuma* larvae, | *Tx.* *moctezuma* larvae initially reduced mosquito pupae, but effectiveness decreased over time | Causality not confirmed (other factors might explain pupal increase) |
| Rawlins et al., 1991 | *Tx. moctezuma* | Field experiment with pre-test/post-test | Introduced *Tx.* *moctezuma* larvae into potential mosquito breeding sites in a village | Significant reduction in adult *Ae. aegypti* population one month after introducing *Tx.* *moctezuma* larvae. | Limited information on pre-introduction mosquito population levels. |
| Tikasinh and Eustace, 1992 | *Tx. moctezuma* | Field experiment with treated (received predator releases) and control | Monitored *Ae. aegypti* populations | *Ae. aegypti* population indices were significantly lower in the village with predator releases compared to the control village | Lack detail on predator release frequency/quantity |
| Tikashing, 1992 | *Tx. moctezuma* | Field experiment | Used containers (drums) with *Ae. aegypti* larvae and introduced *Tx.* *moctezuma* larvae at different densities (1-2, 5, or 10) | *Tx.* *moctezuma* effectively reduced *Ae. aegypti* adult emergence, with higher *Tx.* *moctezuma* density leading to greater reduction and longer prevention | Lack of information about the experiment and potential impact of environmental factors |
| Alomar et al., 2020 | *Tx. rutilus* | factorial design with two manipulated factors | Potentially testing the effects of juvenile exposure to pyriproxyfen and predatory mosquito *Tx. rutilus* on adult *Ae. aegypti* traits | Combining pyriproxyfen exposure with *Tx.* *rutilus* resulted in the strongest inhibition of adult *Ae. aegypti* emergence | Lack of number of replicates used |
| Padgett and Focks 1981 | *Tx. rutilus* | Controlled experiment | Measured consumption (eating) and killing (without eating) of each prey stage by one 4th instar *Tx.* rutilus | *Tx.* *rutilus* preferentially consumed 4th instar prey compared to pupae or 1st instar prey. | Limited to lab setting, prey availability might not reflect natural situations, |
| Goettlw and Adler, 2005 | *Tx. rutilus* | Field experiment | Compare areas with *Tx.* *splendens* releases to control areas without releases | Releasing *Tx.* *splendens* adults can potentially reduce pest mosquito populations | Complete control is unlikely with this method. |
| Jones and Schreiber, 1994 | *Tx.* *rutilus* | Observational study | Observe *Tx.* *splendens* larvae in containers with different prey types (mosquito larvae, Daphnia, brine shrimp) and monitor prey consumption, cannibalism rates, and development success. | *Tx.* *splendens* successfully consumed *Aedes* mosquito | Focused primarily on laboratory or observational research, potentially lacking field experimentation |
| Wood et al., 2022 | *Tx. brevipalpis* | laboratory work with two factors | Metarhizium fungus application, introduction of *Toxorhynchites* predator, and a combined treatment against *Ae. aegypti* | Combined Metarhizium and Toxorhynchites treatment significantly reduced the time it took for *Ae. aegypti* larvae to die compared to individual treatments | Lab setting limits real-world applicability |
| Trpis 1972 | *Tx. brevipalpis* | Observational experiment | Larval development time, prey consumption of *Tx. brevipalpis* were measured. | Predation rate and total prey consumed increased at higher temperatures (30-32°C) compared to lower ones | Unknown if other factors like light or water quality were controlled. |
| Albeny et al., 2011 | *Tx. violaceus* | Observational experiment | *Tx. violaceus* larvae (presumably 4th instar) were introduced with *Ae. aegypti* larvae, and their consumption was monitored over 192 hours (8 days) | Predation of *Ae. aegypti* by *Tx.* *violaceus* larvae increased throughout the experiment, *Ae. aegypti* survival completely dropped by the end of the observation period | Predation rates in a controlled environment might not reflect real-world scenarios, |
| Yasuda 1995 | *Tx. towadensis* | Laboratory experiment | Larvae of *Tx. towadensis* were exposed to different prey densities in controlled conditions. | Prey consumption increased with increasing prey density | Conducted in a controlled environment, potentially not reflecting real-world conditions |
| Albeny‑Simões 2015 | *Tx. theobaldi* | Choice experiment | Gravid *Ae. aegypti* females were offered paired oviposition sites with varying cues. | *Ae. aegypti* preferred oviposition sites with Toxorhynchites predation and dead conspecific larvae | Focused on laboratory settings, potentially not reflecting natural mosquito behavior |
| Russo 1986 | *Tx. amboinensis, Tx. rutilus, Tx. theobaldi, brevipalpis, Tx. splendens* | Comparative experiment with five *Toxorhynchites* mosquito species | Selected multiple *Toxorhynchites* species for comparison and recorded the number of strikes per successful capture for each species. | *Tx.* *amboinensis* and *Tx.* *brevipalpis* had a significantly higher daily capture rate compared to *Tx.* *splendens* and *Tx.* *theobaldi* | Lacked ecological context, as experiments were likely conducted under controlled laboratory conditions |
